# 

**Published:** 1891-09

**Authors:** 


					﻿VOLXI?
SEPTEMBER, 1891.
NO. 97
THE
{HOMEOPATHIC pHY^lClAfl,
A Monthly Journal of Homoeopathic Materia Medica
and Clinical Medicine.
“He is a freeman whom truth makes free, and all are slaves beside."
“Seek the Truth: come whence it may, cost what it wilt,”
EDITED AND PUBLISHED BY
WALTER M. JAMES, M. D.,
AND
George H. Clark, m.d.
PHILADELPHIA :
No. 1125 SPRUCE STREET.
LONDON-
ALFRED HEATH & CO., Sole Agents foe Great Britain,
114 Ebury Street, Eaton Square.
Yearly Subscription, $2.50, invariably in advance. Single numbers, 25 cts.
Entered at the Philadelphia Peet-Office ae Seoond-Clan Mail Matter.
				

